# Hyperglycemic Hyperosmolar State as the Initial Presentation of Wolfram Syndrome: A Common Complication Revealing a Rare Disease—A Case Report

**DOI:** 10.1002/ccr3.71563

**Published:** 2025-11-28

**Authors:** Sushrut Ingawale, Ishaan Shinde, Sai Teja Rachakonda, Ashmita Pandey, Ayushi Shah

**Affiliations:** ^1^ Quinnipiac University—Frank H. Netter MD School of Medicine—St. Vincent's Medical Center Bridgeport Connecticut USA; ^2^ Seth Gordhandas Sunderdas Medical College and King Edward VII Memorial Hospital Mumbai India; ^3^ Alluri Sita Ramaraju Academy of Medical Sciences Eluru India; ^4^ Tribhuwan University Teaching Hospital Kathmandu Nepal; ^5^ SUNY Upstate Medical University Syracuse New York USA

**Keywords:** case report, diabetes insipidus, diabetes mellitus, DIDMOAD syndrome, optic atrophy, Wolfram syndrome

## Abstract

Wolfram syndrome is a rare autosomal recessive disorder characterized by diabetes insipidus, diabetes mellitus, optic atrophy, and deafness (DIDMOAD). We present the case of a 19‐year‐old male with a history of juvenile‐onset non‐autoimmune diabetes mellitus who presented with fever, chills, seizures, altered sensorium, vomiting, and abdominal pain. The patient was treated for a hyperosmolar hyperglycemic state precipitated by gastrointestinal infection with intravenous fluids, antibiotics, and insulin therapy. Physical examination revealed short stature, delayed secondary sexual characteristics, neck rigidity, and bilateral upward plantar reflexes. Further neuroimaging revealed pontine atrophy, partial central diabetes insipidus, and bilateral optic atrophy. Fundoscopy confirmed optic disc pallor and generalized visual field loss. Pure tone audiometry indicated profound bilateral high‐frequency sensorineural hearing loss, and magnetic resonance (MR) urography findings were consistent with a neurogenic bladder. His sensorium and neurological deficits improved within 3 days, and he was later discharged with close follow‐up by a multidisciplinary team. This case highlights the classic presentation of Wolfram syndrome in a young male with diabetes and neurological complications, emphasizing the need for early recognition and multidisciplinary management. Wolfram syndrome poses significant diagnostic challenges due to its varied and progressive symptoms, and this report aims to contribute to the existing knowledge base and create awareness about the condition, its clinical presentation, and the need for a multidisciplinary management approach.

## Summary

Early recognition of Wolfram syndrome in patients with juvenile‐onset, non‐autoimmune diabetes mellitus and multisystem features allows timely referral for multidisciplinary care, genetic counseling, and targeted surveillance, which may reduce complications, improve quality of life, and enhance long‐term outcomes in this rare, progressive condition.

## Graphical abstract

MRI brain revealing features consistent with central diabetes insipidus (Figure A), pontine atrophy (Figure B), and bilateral optic nerve atrophy (Figure C) in a young, non‐autoimmune diabetic patient: imaging clue to Wolfram syndrome.

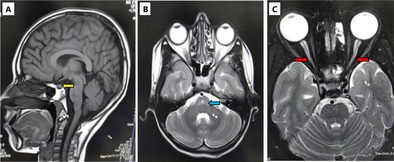

**Abbreviations**
BUN: blood urea nitrogen
CISD2 gene: CDGSH iron sulfur domain 2 gene
DIDMOAD: diabetes insipidus, diabetes mellitus, optic atrophy, and deafness
DKA: diabetic ketoacidosis
DM: diabetes mellitus
GLP‐1: glucagon‐like peptide‐1
HHS: hyperglycemic hyperosmolar state
ICD‐11: International Classification of Diseases, 11th revision
MR urography: magnetic resonance urography
MRI: magnetic resonance imaging
WBC: white blood cell
WFS1: Wolfram syndrome type 1
WFS2: Wolfram syndrome type 2

## 1 Introduction

Wolfram syndrome, also known as DIDMOAD syndrome, is a rare autosomal recessive disorder characterized by Diabetes Insipidus (DI), Diabetes Mellitus (DM), Optic Atrophy (OA), and Deafness (D). It results from mutations in the *WFS1* or *CISD2* genes, leading to progressive neurodegeneration and multisystem involvement [1]. Wolfram syndrome type 1 is a rare type of DM and has been included in subcategory 5A16.1 of the International Classification of Diseases (ICD‐11) [2]. Diagnosis is challenging and requires a high index of suspicion followed by a multidisciplinary team for symptom management. However, there is no definitive treatment for this condition. Wolfram syndrome has a poor prognosis and is associated with premature death at the mean age of 30 years (25–49 years) due to respiratory failure, often as a result of brainstem involvement [3, 4]. There is limited literature on the presentation and management of Wolfram syndrome, and this report aimed to expand the existing knowledge base. Careful clinical follow‐up and supportive management can help relieve severe, progressive symptoms, while clinical vigilance during acute presentations can aid in identifying rare underlying diseases, as demonstrated in this case.

## 2 Case History and Examination

A 19‐year‐old male of South Asian ethnicity, born to a non‐consanguineous marriage, presented to a tertiary care center with fever and chills for the past 20 days. The patient had multiple episodes of generalized tonic–clonic seizures over the past 2 days, associated with upward rolling of the eyes and biting of the tongue, followed by an altered sensorium. He also experienced two episodes of non‐bilious vomiting during the 2 days associated with diffuse abdominal pain of a dull, aching nature.

The patient was a known case of juvenile‐onset, non‐autoimmune diabetes mellitus since the age of nine and was non‐compliant with insulin. The patient had a history of recurrent episodes of loose stools since childhood, along with a history of nocturia and incomplete bladder evacuation. The patient's family history and psychosocial history were non‐contributory.

**FIGURE 1:**
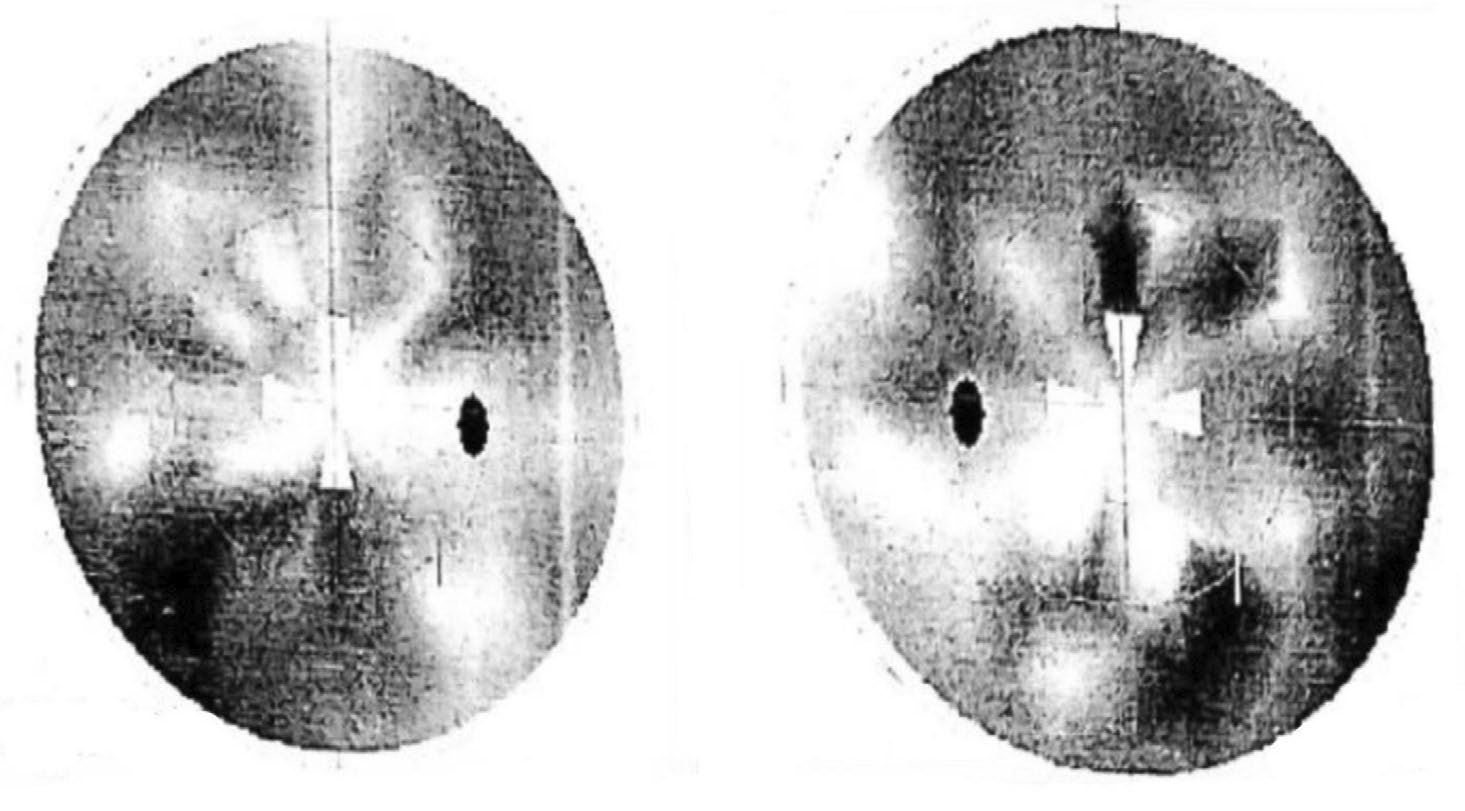
Visual testing revealing bilateral optic disc pallor, suggestive of bilateral optic atrophy.

At the time of presentation, vitals were: temperature 37.0°C, heart rate 114 beats per minute with a regular normal pulse, blood pressure 94/70 mmHg, peripheral oxygen saturation 98% on room air, and no jugular venous distension. These vitals normalized after adequate fluid resuscitation. On physical examination, the patient appeared dehydrated with dry mucosal membranes. The patient was irritable and had an altered sensorium. The neurological examination revealed bilateral neck rigidity and a sluggish pupillary response. The patient had an upward plantar reflex bilaterally, with normal muscular tone throughout the limb. On detailed examination from an endocrine standpoint, he was also noted to have a short stature (height 155 cm) with a few features of poorly developed secondary sexual characteristics, like scanty and thin body hair, and below‐average muscle development. But, pubic and axillary hair were present; voice was normal. He had attained his puberty at the appropriate age and no delays were reported or noticed. On ocular examination, the patient had optic disc pallor in both eyes on fundoscopy (as seen in Figure 1) and generalized visual field loss on perimetry, along with compromised color vision, consistent with bilateral optic atrophy.

## 3 Differential Diagnosis, Investigations and Treatment

### 3.1 Differential Diagnosis

Although the patient was a known case of Diabetes Mellitus, atypical features such as optic atrophy, diabetes insipidus, neurogenic bladder, delayed puberty, and sensorineural hearing loss suggested a multisystem disorder. Several differential diagnoses were considered, as mentioned in Table 1.

**TABLE 1:** Differential diagnosis and reasons for exclusion.

Differentials	Key features	Reason for exclusion
Maternally inherited diabetes and deafness (MIDD)	Diabetes with sensorineural hearing loss; mitochondrial inheritance	No maternal inheritance, no family history, no macular involvement
Leber hereditary optic neuropathy (LHON)	Acute central vision loss; maternal inheritance	Patient had diabetes, DI, and deafness; no maternal history
Alström syndrome	Diabetes, hearing loss, cone‐rod dystrophy, obesity, cardiomyopathy	No obesity or cardiomyopathy
Friedreich ataxia	Ataxia, cardiomyopathy, ±diabetes	No ataxia or cardiomyopathy; optic atrophy + deafness not typical
Wolfram syndrome	Juvenile diabetes, optic atrophy, DI, deafness, neurogenic bladder	Patient's features strongly consistent → Final diagnosis

*Note:* Comparison of alternative syndromes considered in this case, with their key clinical features and the reasons for their exclusion. The final diagnosis of Wolfram Syndrome was established based on the patient's multisystem involvement.

### 3.2 Investigations

The patient's initial laboratory evaluation revealed significant metabolic derangements. Serum glucose was markedly elevated at 863 mg/dL (reference: 70–110 mg/dL), indicating severe hyperglycemia, and serum osmolarity was elevated at 366 mOsm/kg (275–295 mOsm/kg), consistent with a hyperosmolar state. HbA1c was 14.2% (< 6.5%), reflecting poor long‐term glycemic control. Arterial blood gas showed a pH of 7.43 (7.35–7.45), ${\mathrm{HCO}}_{3}^{-}$ of 20.6 mmol/L (22–28 mmol/L), and pCO_2_ of 31 mmHg (35–45 mmHg), indicating compensated metabolic acidosis. The patient was hypoxemic with a pO_2_ of 46 mmHg (80–100 mmHg) and a SaO_2_ of 83% (> 95%). Electrolyte analysis showed hypernatremia with sodium at 154 mmol/L (135–145 mmol/L), low–normal potassium at 3.5 mmol/L (3.5–5.0 mmol/L), and mildly elevated chloride at 107 mmol/L (98–106 mmol/L). Anion gap was significantly elevated at 43.5 mmol/L (8–16 mmol/L). Renal function was impaired with a creatinine of 2.2 mg/dL (0.6–1.3 mg/dL) and BUN of 27 mg/dL (7–20 mg/dL), consistent with acute kidney injury. White blood cell count was elevated at 31.8 × 10^9^/L (4–11 × 10^9^/L), indicating leukocytosis. Urinalysis revealed glycosuria (4+) but no urinary ketones, which is not consistent with diabetic ketoacidosis (DKA). Due to a lack of resources, the serum ketone levels could not be tested. However, the severity of the osmolar gap, non‐acidotic blood pH, and absence of urinary ketones on multiple settings made HHS more likely than DKA.

### 3.3 Other Investigations

**FIGURE 2:**
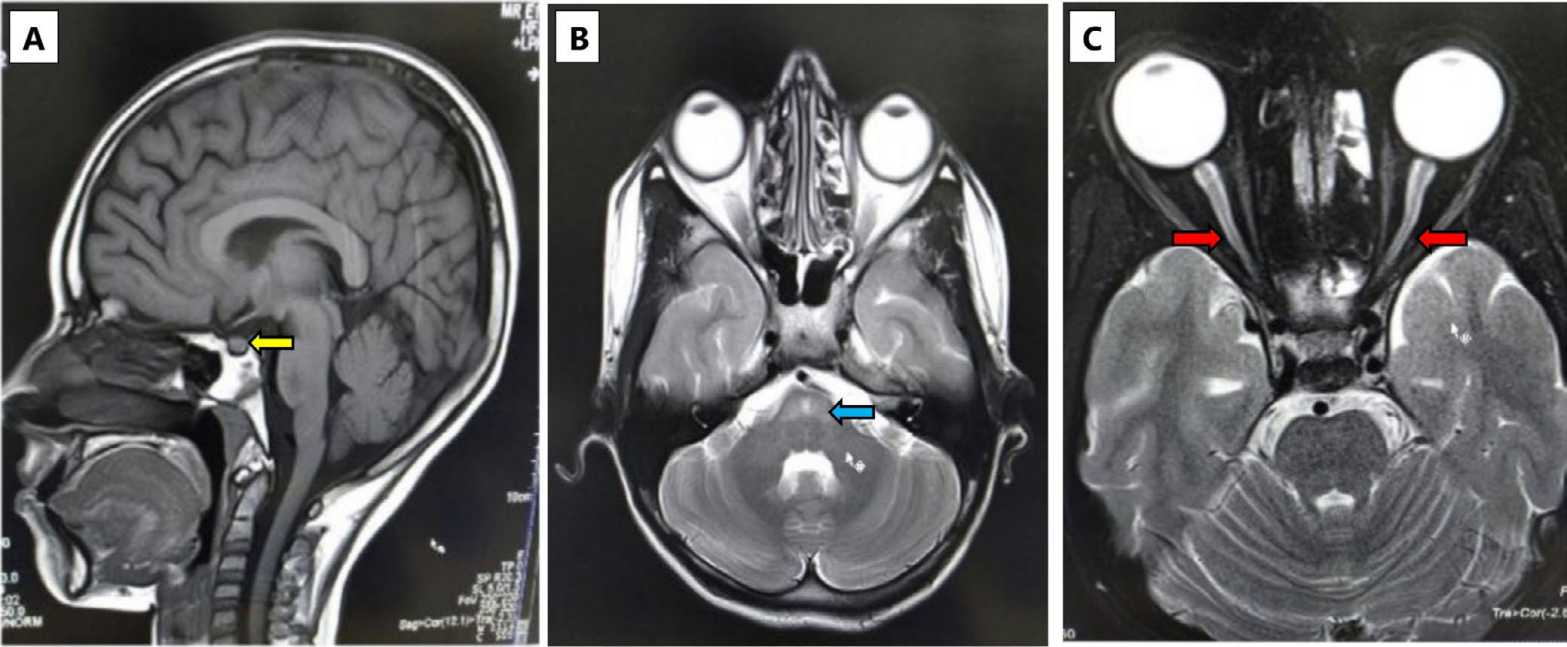
Magnetic resonance imaging brain revealing: (A) (T1 sequence, sagittal view) loss of the normal bright signal (yellow arrow) in the posterior pituitary region; (B) (T2 sequence, transverse view) showing bright, cruciform signals (blue arrow) in the pontine region; and (C) (orbital cuts) showing bilateral optic nerve atrophy (red arrows).

**FIGURE 3:**
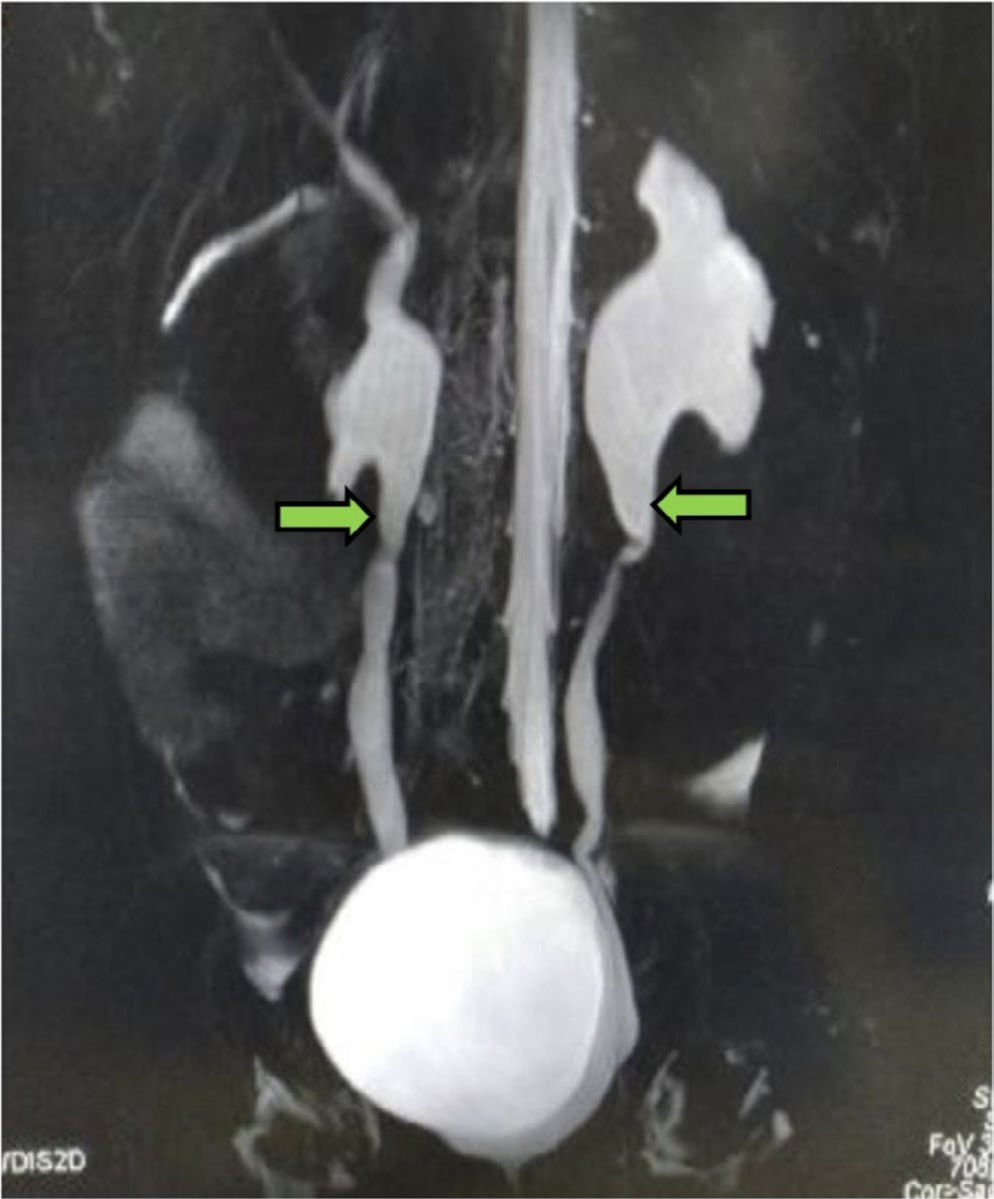
Magnetic resonance urography showing bilateral hydronephrosis and hydroureter (green arrows) with a distended bladder.

Ophthalmologic evaluation revealed optic disc pallor and corresponding visual field loss (Figure 1). Brain MRI demonstrated features consistent with central diabetes insipidus (Figure 2A), pontine atrophy (Figure 2B), and bilateral optic nerve atrophy (Figure 2C). Audiological assessment using pure‐tone audiometry indicated bilateral high‐frequency sensorineural hearing loss. Urological imaging via MR urography showed evidence of hydronephrosis, hydroureter, and a markedly distended bladder (Figure 3).

### 3.4 Treatment

The patient was treated for HHS with intravenous fluids, insulin, and antibiotics. Sensorium improved by day 3, and metabolic parameters normalized by day 12. Persistent optic atrophy remained, as irreversible. He was discharged with referral for multidisciplinary follow‐up, including endocrinology, neurology, ophthalmology, audiology, and urology.

## 4 Outcome and Follow‐Up

On the third day of treatment, the patient's sensorium improved, and subsequent neurological examinations returned to normal. However, optic atrophy persisted, as this condition is irreversible. By the twelfth day of treatment, blood glucose levels, serum osmolarity, electrolyte concentrations, and white blood cell counts had normalized to within physiological reference ranges. Based on the combination of endocrine, neurological, and urological findings, Wolfram syndrome was clinically diagnosed. Genetic testing was not conducted due to resource limitations in the clinical setting. The patient was referred to the Department of Endocrinology for further evaluation and discharged with close follow‐up for multidisciplinary management.

## 5 Discussion

Genetic diseases diagnosed in adulthood require special attention. Wolfram syndrome is a rare and complicated syndrome that necessitates a multidisciplinary approach. The syndrome has two subtypes: Wolfram syndrome 1 (WFS1), associated with the *WFS1* gene on chromosome 4p16.1, which encodes wolframin, and Wolfram syndrome 2 (WFS2), related to the *CISD2* gene on chromosome 4q24. Both proteins are involved in calcium homeostasis and the regulation of endoplasmic reticulum oxidative stress. Mutations in WFS1 lead to multisystem neurodegeneration and account for most cases of Wolfram syndrome, with over 200 variants identified to date, predominantly in exon 8. Wolframin is primarily expressed in the pancreas, nerve cells, eyes, inner ear, liver, and kidneys [1].

This condition is seen in about 1 in 500,000 children and is found to be associated with consanguineous marriage. The pathophysiological basis of this condition is dysfunction of the endoplasmic reticulum due to WFS1 mutation, leading to the accumulation of misfolded proteins, causing severe stress, presenting clinically as degeneration of neurons and beta cells of the pancreas, in addition to a constellation of systemic symptoms. In a study by Ray et al., 93.2% of patients with Wolfram syndrome had diabetes mellitus [5]. Its life expectancy is 39 years (range: 25–49 years), with death occurring due to respiratory failure secondary to brainstem atrophy [6]. In Wolfram syndrome, diabetes mellitus typically develops in late childhood (6–10 years), optic atrophy in early adolescence (10–15 years), diabetes insipidus in late adolescence (14–20 years), sensorineural hearing loss in late adolescence (16–20 years), and ataxia in early adulthood (15–25 years) [7]. This case report effectively highlights the classical presentation and diagnostic features of Wolfram syndrome.

Our patient also demonstrated renal involvement (neurogenic bladder) and gonadal involvement (delayed secondary sexual characteristics), which are less frequently reported manifestations of Wolfram syndrome. Differentiating WFS1 (wolframin mutation), usually presenting with diabetes mellitus and optic atrophy, from WFS2 (CISD2 mutation), often associated with additional gastrointestinal ulceration and bleeding, remains important in guiding counseling and prognosis.

This case reinforces that even common acute presentations, such as hyperosmolar hyperglycemic state (HHS), can serve as pivotal diagnostic opportunities for uncovering rare multisystem disorders like Wolfram syndrome. Early recognition of atypical features in young patients with presumed type 1 or type 2 diabetes, particularly the coexistence of optic atrophy, diabetes insipidus, or neurogenic bladder, should raise clinical suspicion for Wolfram syndrome, even before confirmatory genetic testing is available. Awareness of this constellation of findings can prompt timely diagnostic evaluation, facilitate appropriate genetic counseling, and enable early multidisciplinary interventions aimed at preserving neurologic and visual function. Ultimately, this case highlights the critical importance of maintaining diagnostic vigilance and integrating systemic findings into acute diabetes care to improve long‐term outcomes in rare metabolic disorders.

Although the current management of Wolfram syndrome remains supportive, emerging strategies targeting endoplasmic reticulum calcium homeostasis, protein folding, and oxidative stress, such as dantrolene, chemical chaperones, GLP‐1 receptor agonists, and gene therapy, hold promise for modifying disease progression [6].

## 6 Conclusion

This case highlights the importance of early clinical recognition of Wolfram syndrome in resource‐limited settings, where genetic testing may not be available. Vigilance is essential in young patients with juvenile‐onset, non‐autoimmune diabetes mellitus and multisystem involvement, as timely monitoring and multidisciplinary management can significantly improve quality of life. Genetic counseling should also be offered to affected families, particularly in populations with higher consanguinity rates, to support early recognition and informed reproductive decisions.

## Author Contributions

**Sushrut Ingawale:** conceptualization, formal analysis, investigation, resources, supervision, writing – original draft, writing – review and editing. **Ishaan Shinde:** formal analysis, investigation, writing – original draft. **Sai Teja Rachakonda:** formal analysis, investigation, writing – original draft. **Ashmita Pandey:** formal analysis, investigation, writing – original draft, writing – review and editing. **Ayushi Shah:** supervision, writing – review and editing.

## Funding

The authors have nothing to report.

## Disclosure

The authors have nothing to report.

## Consent

Written informed consent was obtained from the patient for publication of this case report and accompanying images.

## Conflicts of Interest

The authors declare no conflicts of interest.

## Data Availability

The de‐identified clinical data supporting the findings of this case report are available upon reasonable request, subject to institutional and ethical approval to protect patient confidentiality.
